# Exploring the diagnostic effectiveness for myocardial ischaemia based on CCTA myocardial texture features

**DOI:** 10.1186/s12872-021-02206-z

**Published:** 2021-08-31

**Authors:** Hengyu Zhao, Lijie Yuan, Zhishang Chen, Yuting Liao, Jiangzhou Lin

**Affiliations:** 1 Xiamen Cardiovascular Hospital Xiamen University, Xiamen, 361006 Fujian China; 2 Department of Molecular Biology, Xiamen Medical College, Xiamen, China; 3 GE Healthcare, Guangzhou, China; 4 Key Laboratory of Functional and Clinical Translational Medicine, Fujian Province University, Xiamen Medical College, Xiamen, China; 5 Xiamen Key Laboratory of Precision Medicine for Cardiovascular Disease, Xiamen, China

**Keywords:** Coronary atherosclerosis, Coronary CT angiography, Myocardial ischaemia, Texture features

## Abstract

**Background:**

To explore the characteristics of myocardial textures on coronary computed tomography angiography (CCTA) images in patients with coronary atherosclerotic heart disease, a classification model was established, and the diagnostic effectiveness of CCTA for myocardial ischaemia patients was explored.

**Methods:**

This was a retrospective analysis of the CCTA images of 155 patients with clinically diagnosed coronary heart disease from September 2019 to January 2020, 79 of whom were considered positive (myocardial ischaemia) and 76 negative (normal myocardial blood supply) according to their clinical diagnoses. By using the deep learning model-based CQK software, the myocardium was automatically segmented from the CCTA images and used to extract texture features. All patients were randomly divided into a training cohort and a test cohort at a 7:3 ratio. The Spearman correlation and least absolute shrinkage and selection operator (LASSO) method were used for feature selection. Based on the selected features of the training cohort, a multivariable logistic regression model was established. Finally, the test cohort was used to verify the regression model.

**Results:**

A total of 387 features were extracted from the CCTA images of the 155 coronary heart disease patients. After performing dimensionality reduction with the Spearman correlation and LASSO, three texture features were selected. The accuracy, area under the curve, specificity, sensitivity, positive predictive value and negative predictive value of the constructed multivariable logistic regression model with the test cohort were 0.783, 0.875, 0.733, 0.875, 0.650 and 0.769, respectively.

**Conclusion:**

CCTA imaging texture features of the myocardium have potential as biomarkers for diagnosing myocardial ischaemia.

**Supplementary Information:**

The online version contains supplementary material available at 10.1186/s12872-021-02206-z.

## Background

Coronary atherosclerotic heart disease (CHD), also referred to as coronary heart disease, is one of the leading causes of death and disability worldwide [1–3]. It is caused by atherosclerosis of important blood vessels arising from internal and/or external factors, resulting in stenosis or occlusion of the lumen and eventually myocardial ischaemia or necrosis (also known as ischaemic heart disease). The main clinical symptom of myocardial ischaemia is chest pain, and clinical treatment includes medication, percutaneous coronary intervention, coronary artery bypass grafting and so on. The early diagnosis of myocardial ischaemia in patients with CHD is crucial for the selection of appropriate treatment.

Coronary CT angiography (CCTA) is a non-invasive imaging technique that can clearly display the anatomy of the coronary arteries. In addition, it has advantages in evaluating coronary artery stenosis, atherosclerotic plaque, calcification, and cardiac function (based on non-invasive coronary fractional flow reserve (FFR)) [4–7]. Therefore, it is considered a routine examination method for patients with suspected CHD. However, it is difficult to assess myocardial ischaemia by visual changes in myocardial tissue density on CCTA images, as conventional CCTA images show limited contrast. Furthermore, the assessment of haemodynamics by coronary artery lesions displayed on CCTA images remains unclear since there is no significant relationship between arterial stenosis and blood flow changes in the myocardium [8, 9]. Therefore, a combination of different examinations for the diagnosis of myocardial ischaemia is needed (stress single-photon emission computed tomography/positron emission tomography (SPECT/PET), stress cardiac magnetic resonance (CMR), or invasive FFR) [10].

Recently, radiomics has shown great potential for various cancers in terms of pathological classification, the assessment of tumour metastasis, clinical outcomes and gene expression [11–13]. Radiomics can be used to non-invasively extract a large number of high-level quantitative features from medical images, especially parameters that are invisible to the naked eye or cannot be quantified through routine analysis. If the imaging features of CCTA can be used to assess whether myocardial ischaemia is present in CHD patients, they could have a positive impact on the technology of CCTA and the clinical diagnosis and treatment of CHD patients. Therefore, the purpose of this study was to explore the use of CCTA imaging features to evaluate myocardial ischaemia in patients with CHD.

## Methods

### Materials

All patients with a diagnosis of myocardial ischaemia with available CCTA images between September 1, 2019, and January 1, 2020, at Xiamen Cardiovascular Hospital Xiamen University in Fujian Province were retrospectively included in this study. The diagnosis of myocardial ischaemia was based on clinical diagnosis and/or single photon emission computed tomography (SPECT) imaging findings. To ensure that the corresponding clinical and imaging data of the included patients were suitable for the purposes of our research, we established the following inclusion criteria: (1) The interval between CCTA image acquisition and myocardial ischaemic diagnosis was less than two weeks. (2) The absence of a combination of other heart diseases, a history of coronary artery bypass grafting or coronary stent implantation, as these conditions may affect the results. Finally, a total of 79 patients with clinically diagnosed myocardial ischaemia were enrolled. The study also included 76 age-matched healthy controls with CCTA scans performed during the same time period.

The following patient information was collected: demographic data, including sex, age and weight; clinical data, including history of hypertension, history of hyperlipidaemia, history of diabetes, smoking status, clinical characteristics and myocardial enzymes; and imaging data, including electrocardiography and CCTA.

### Diagnosis of myocardial ischaemia

**Fig. 1:**
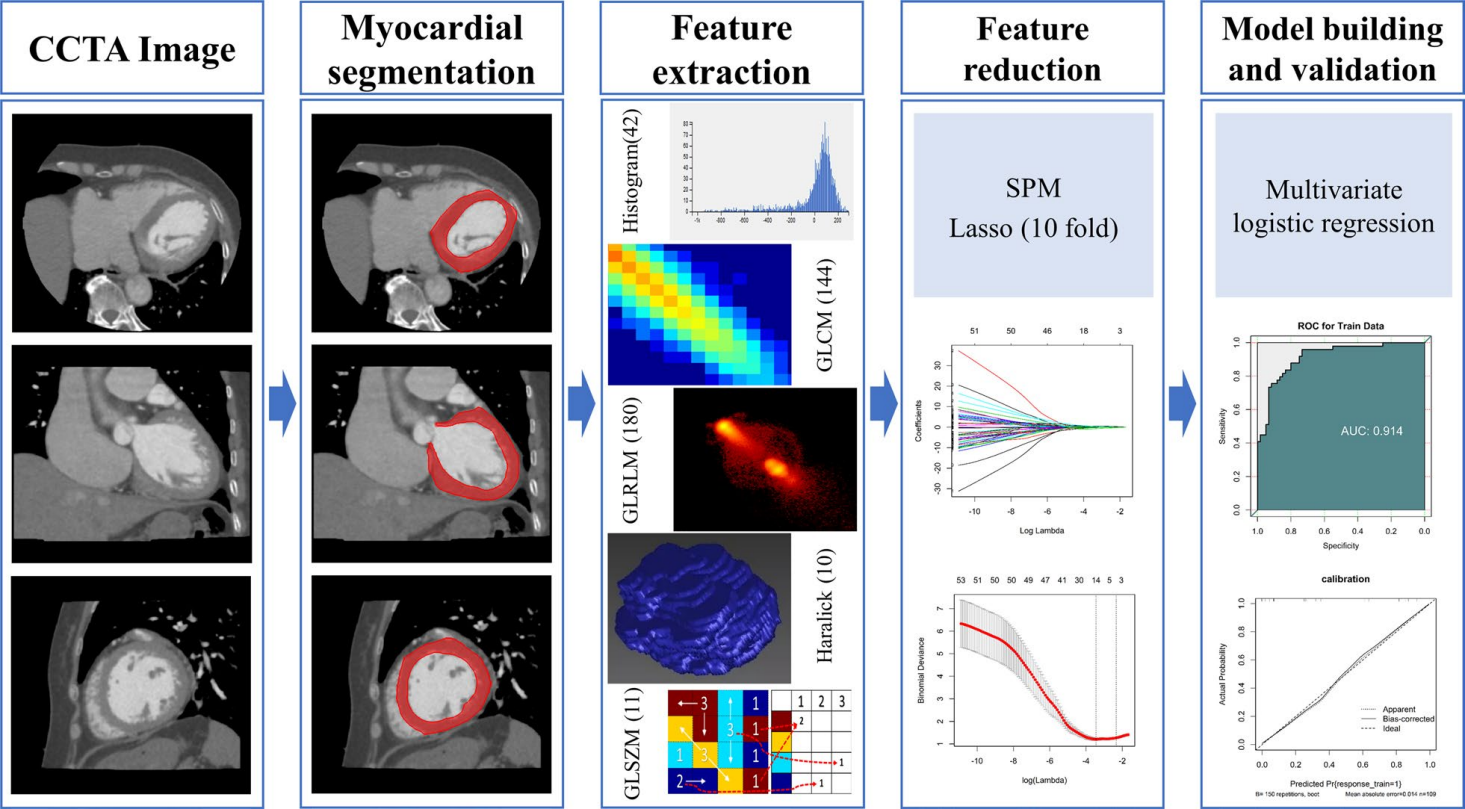
Workflow of the construction of the myocardial ischaemia model

In this study, myocardial ischaemia was diagnosed based on stress SPECT imaging and/or the patient’s clinical characteristics, electrocardiogram and myocardial enzymes (Fig. 1). The diagnosis process was as follows: the patient’s clinical characteristics, electrocardiogram and myocardial enzymes were first judged. If the patient was not suspected of having myocardial ischaemia, she was initially enrolled in the normal myocardial blood supply group (negative group) of this study. If the patient had signs of myocardial ischaemia, she was subjected to a stress SPECT scan for diagnosis. Patients diagnosed with myocardial ischaemia according to stress SPECT were initially enrolled in the myocardial ischaemia group (positive group) of this study.

The stress SPECT scan was performed with a D-SPECT Cardiac Scanner System (Spectrum Dynamics Medical Ltd. Israel). The patient received an intravenous injection of adenosine disodium triphosphate (Tianjin Jinyao Pharmaceutical Co., Ltd. China) at a rate of 0.14 mg/kg.min, which was completed continuously within 6 min. Three minutes after the start of the injection, the patient received a simultaneous bolus injection of 99mTC-MIBI (Fuzhou Jiayi Pharmaceutical Co., Ltd. China) at a dose of 24.0 mCi via another intravenous channel. Approximately 1 h later, the patient underwent stress-gated myocardial tomography.

### CCTA scans

**Fig. 2:**
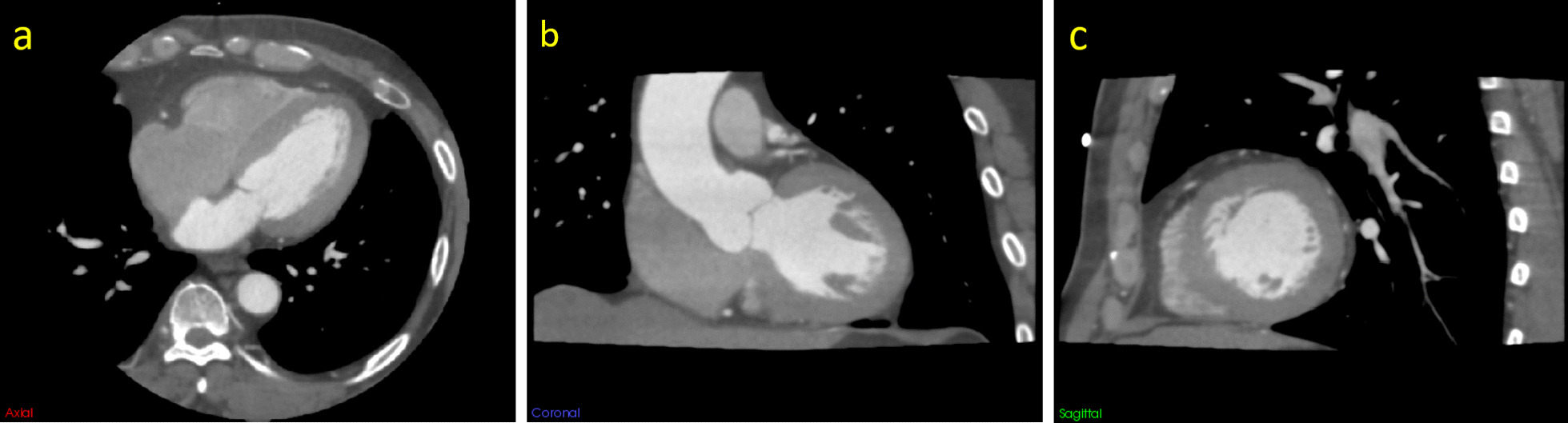
A 56-year-old male patient with myocardial ischaemia. **a** Axial image, **b** coronal image, and **c** sagittal image. The window level was set to 100 HU, and the window width to 800 HU

**Fig. 3:**
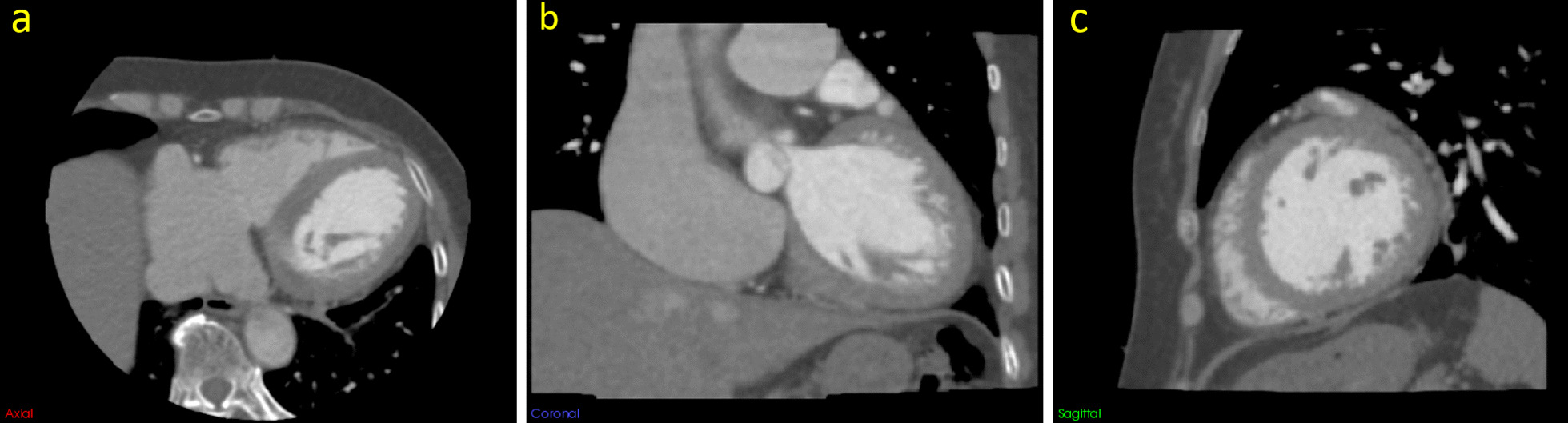
A 61-year-old female patient without myocardial ischaemia. **a** Axial image, **b** coronal image, and **c** sagittal image. The window level was set to 100 HU, and the window width to 800 HU

All patients received a CCTA scan on an empty stomach with a 560-slice multi-slice spiral cardiovascular CT device (CardioGraphe™; GE Healthcare). The scan range was from the tracheal crest to the bottom of the heart. The scanning parameters were as follows: tube voltage 120 kVp; tube current 50 mA; CT rotation time 0.24 s; and reconstruction layer thickness 0.5 mm. The contrast agent was injected from a vein through a high-pressure syringe (Salient; Imaxeon Pty Ltd.), and scanning started 5 s after reaching the trigger threshold. The contrast medium used was iohexol injection (Omnipaque, 350 mg I/ml, GE Healthcare) with the following injection programme: duration of coronary drug injection 12 s; heart rate ≥ 75 beats/min; every additional 5 beats/min, the rate was increased by 0.2 ml/s, and the amount of medicine was increased similarly. A 30 ml dose of saline was injected at the same rate as the contrast. The CCTA scans were performed in accordance with the 2016 Society of Cardiovascular Computed Tomography (SCCT) guidelines for the performance and acquisition of coronary computed tomographic angiography, and the scan parameter settings followed the "as low as reasonably achievable" (ALARA) principle [14]. The contrast media injection protocol is shown in Table 1 in Additional file 1. Figures 2 and 3 show the CCTA images of patient with and without myocardial ischaemia, respectively.

### Myocardial segmentation and feature extraction

The CCTA images were imported into CQK software (CT Coronary Artery Quantitative Analysis Kit, CQK, GE Healthcare, China) for automatic myocardium segmentation and feature extraction. A radiological expert with 10 years of experience verified the segmentation results. A total of 387 features were extracted from the three-dimensional segmented myocardium, including histogram parameters (42 features), grey level cooccurrence matrix features (144), Haralick feature s(10), grey level run-length matrix features (180) and grey level size zone matrix features (11). The corresponding formula and meaning of each feature are detailed in Additional file 2.

### CCTA myocardial features

The dataset was randomly assigned in a 7:3 ratio to either a training cohort or a test cohort. All images in the training cohort were used to train the predictive model, while those in the test cohort were used to independently evaluate the model performance. Before the analyses, variables with zero variance were excluded. Then, missing and outlier values were replaced by the median, and finally, the data were standardized.

### Feature selection and machine learning model construction

Feature selection was performed by using Spearman correlation analysis (SPM) and the least absolute shrinkage and selection operator (LASSO). A Spearman correlation coefficient between two features of ≥ 0.9 was considered to indicate a relevant correlation, and only one of the two features was selected randomly to reduce feature redundancy. The LASSO method was used to further select features with penalty parameter tuning that was conducted by tenfold cross-validation based on minimum criteria. Finally, we obtained the optimal feature subset. A multivariable logistic regression model was constructed based on the optimal feature subset from the training cohort. Multivariable logistic regression is a machine learning method that analyses the relationship between multiple variables and two-classification dependent variables and solves the problem of binary classification (0 or 1). Then, the optimal features from the test cohort were imported into the constructed model to verify its discriminative performance. To visualize and validate the multivariable logistic regression model, we built a radiomics nomogram.

### Statistical power calculation

For the sample size of test cohort, Shein-Chung Chow and colleagues [15] introduced a sample size estimation method for clinical research. According to their book, the sample size calculation to test whether the means of two groups are significantly different is as follows.

Let the positive and negative groups be $A$ and $B$ and $\mu$ represent the mean of the radiomics features in each group, with the hypotheses of interest being:

$${H}_{0}:{\mu }_{A}-{\mu }_{B}=0$$

$${H}_{1}:{\mu }_{A}-{\mu }_{B}\ne 0$$

The statistical powers are calculated, respectively, as:

$${N}_{A}=\left(\frac{{n}_{A}+{n}_{B}}{{n}_{B}}\right){\left(\sigma \frac{{z}_{1-\alpha /2}+{z}_{1-\beta }}{{\mu }_{A}-{\mu }_{B}}\right)}^{2}$$

$${N}_{B}=\left(\frac{{n}_{A}+{n}_{B}}{{n}_{A}}\right){\left(\sigma \frac{{z}_{1-\alpha /2}+{z}_{1-\beta }}{{\mu }_{A}-{\mu }_{B}}\right)}^{2}$$

$$1-\beta =\Phi \left(z-{z}_{1-\alpha /2}\right)+\Phi \left(-z-{z}_{1-\alpha /2}\right),\mathrm{ z}=\frac{{\mu }_{A}-{\mu }_{B}}{\sigma \sqrt{\frac{1}{{n}_{A}}+\frac{1}{{n}_{B}}}}$$

where $n$ is the sample size in the training cohort, $N$ is the sample size in the test cohort, $\Phi$ is the standard normal distribution function, $\alpha$ is the type I error, $\beta$ is the type II error, $1-\beta$ is the power, and ${\sigma }^{2}$ is the variance of the covariate.

### Statistical analysis

In this study, the patients were divided into a myocardial ischaemia group and a normal myocardial blood supply group. Continuous variables with a normal distribution are expressed as the mean ± standard deviation, and Student's t-test was utilized for comparisons between two groups. Continuous variables with nonnormal distributions are expressed as medians (interquartile ranges), and the Mann–Whitney *U* test was utilized for comparisons between two groups. Categorical variables are expressed as frequencies (percentages) and were compared with the chi-square test.

All statistical analyses for the present study were performed with R software (version 3.5.1, http://www.r-project.org/). A two-tailed *p*-value < 0.05 indicated statistical significance. The SPM method and multivariable logistic regression model were performed with the “stats” package in R software, and the LASSO method was performed with the “glmnet” package. Receiver operating characteristic (ROC) curves were generated to determine the performance of the machine learning model with the “pROC” package, and the accuracy (ACC), sensitivity, specificity and area under the curve (AUC) were calculated with the “ReprotROC” package. The nomogram was generated with the “rms” package.

## Results

### Clinical data analysis

According to the evaluation criteria for myocardial ischaemia, the 155 coronary heart disease patients enrolled in this study included 79 patients with myocardial ischaemia (positive patients, 63.62 ± 11.61 years) and 76 patients with normal myocardial blood supply (negative patients, 55.37 ± 11.06 years). There were 59 and 47 male patients in the positive group and negative group, respectively, with p = 0.086.

### Feature extraction

**Fig. 4:**
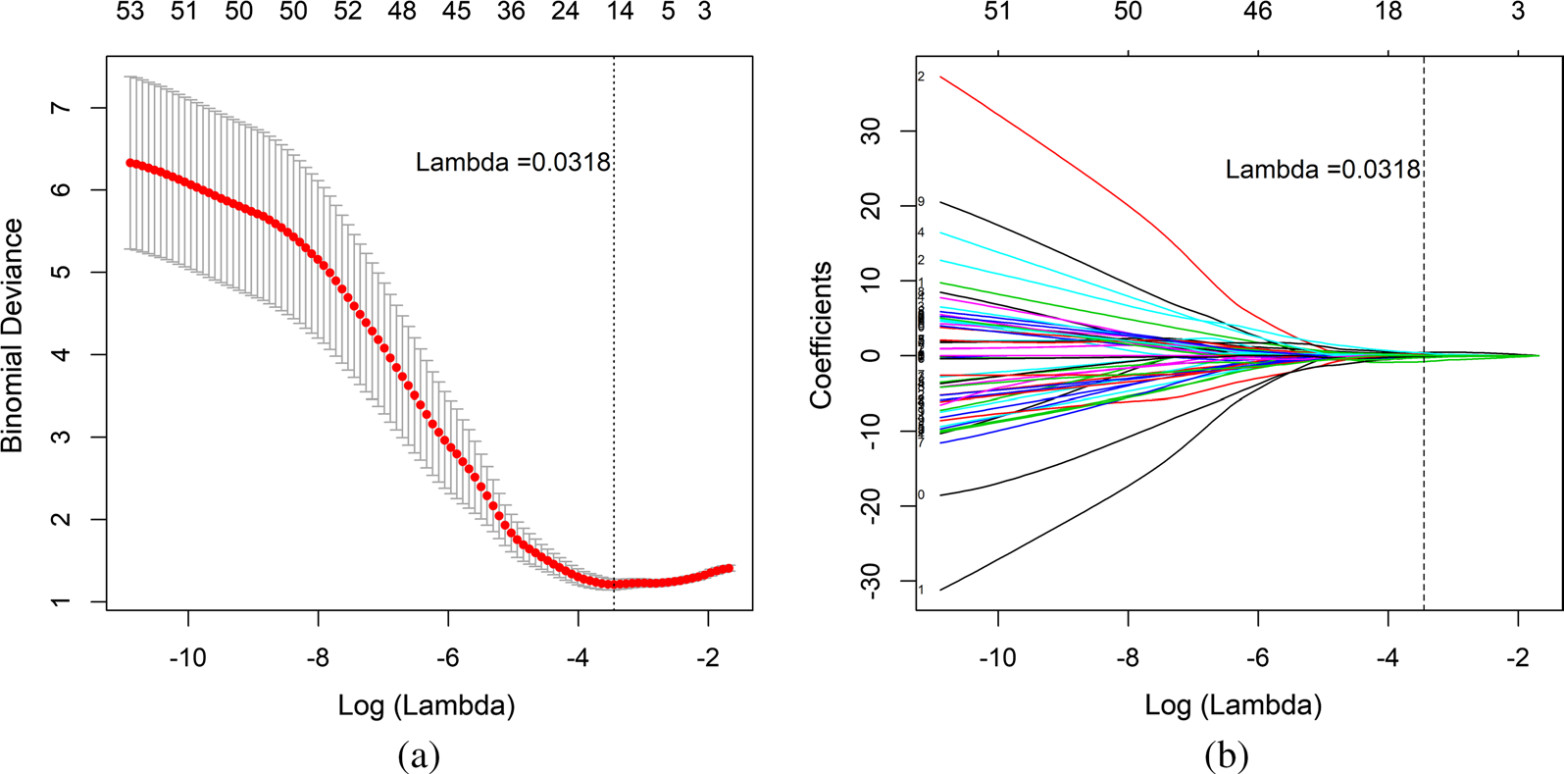
Feature selection and dimension reduction. **a** Ten-fold cross-validation of the LASSO analysis was applied to acquire the most valuable features when the minimum lambda value was reached. **b** The regression coefficients from LASSO

A total of 155 patients were included in this study. For each patient, 387 features were extracted. After using SPM, 68 features remained. Then, 14 features were selected by utilizing the LASSO method (Fig. 4). The 14 texture features of the training cohort were used to build a multivariable logistic regression model. In this model, a weighting factor is assigned based on the impact of each feature on the predicted results. The 14 texture features and their corresponding coefficients in the multivariable logistic regression model are listed in Table 1. The Radscore was calculated by summing the intercept and the product of the feature values and the corresponding coefficients and can be expressed as follows:

**Table 1:** Remaining features and the corresponding coefficients in multivariate logistic regression model

Texture features	Estimate
(Intercept)	− 1.022
RelativeDeviation	0.886
VoxelValueSum	− 1.696
histogramEntropy	− 0.404
skewness	− 0.228
Correlation_AllDirection_offset7_SD	0.440
GLCMEntropy_angle45_offset7	0.096
HaralickCorrelation_angle90_offset7	0.065
RunLengthNonuniformity_AllDirection_offset7_SD	1.625
ShortRunEmphasis_AllDirection_offset4_SD	0.942
ShortRunEmphasis_angle45_offset7	1.508
ShortRunLowGreyLevelEmphasis_AllDirection_offset1_SD	0.820
ShortRunLowGreyLevelEmphasis_AllDirection_offset7_SD	− 4.241
SizeZoneVariability	− 0.531
LowIntensitySmallAreaEmphasis	− 0.252

Radscore = 0.886*RelativeDeviation + (− 0.696)*VoxelValueSum + (0.404)*histogramEntropy + (− 0.228)*skewness + (0.440)*Correlation_AllDirection_offset7_SD + (0.096)*GLCMEntropy_angle45_offset7 + (0.065)*HaralickCorrelation_angle90_offset7 + (1.625)*RunLengthNonuniformity_AllDirection_offset7_SD + (0.942)*ShortRunEmphasis_AllDirection_offset4_SD + (1.508)*ShortRunEmphasis_angle45_offset7 + (0.820)*ShortRunLowGreyLevelEmphasis_AllDirection_offset1_SD + (− 0.241)*ShortRunLowGreyLevelEmphasis_AllDirection_offset7_SD + (− 0.531)*SizeZoneVariability + (− 0.252)* LowIntensitySmallAreaEmphasis.

### Machine learning model

The selected 14 CCTA features were utilized to construct the multivariable logistic regression model for the diagnosis of myocardial ischaemia.

**Fig. 5:**
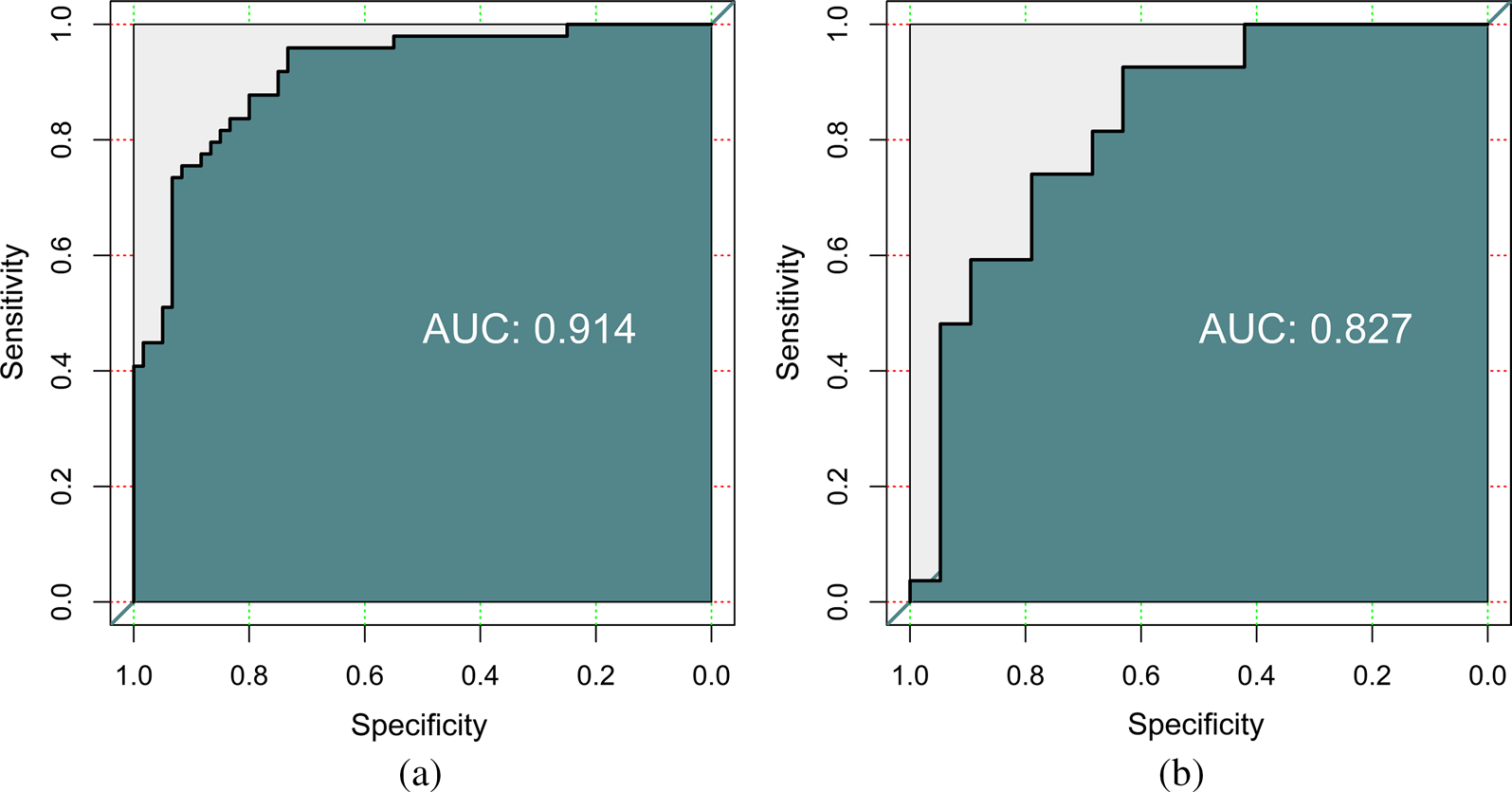
ROC curves of the training cohort (**a**) and test cohort (**b**)

**Fig. 6:**
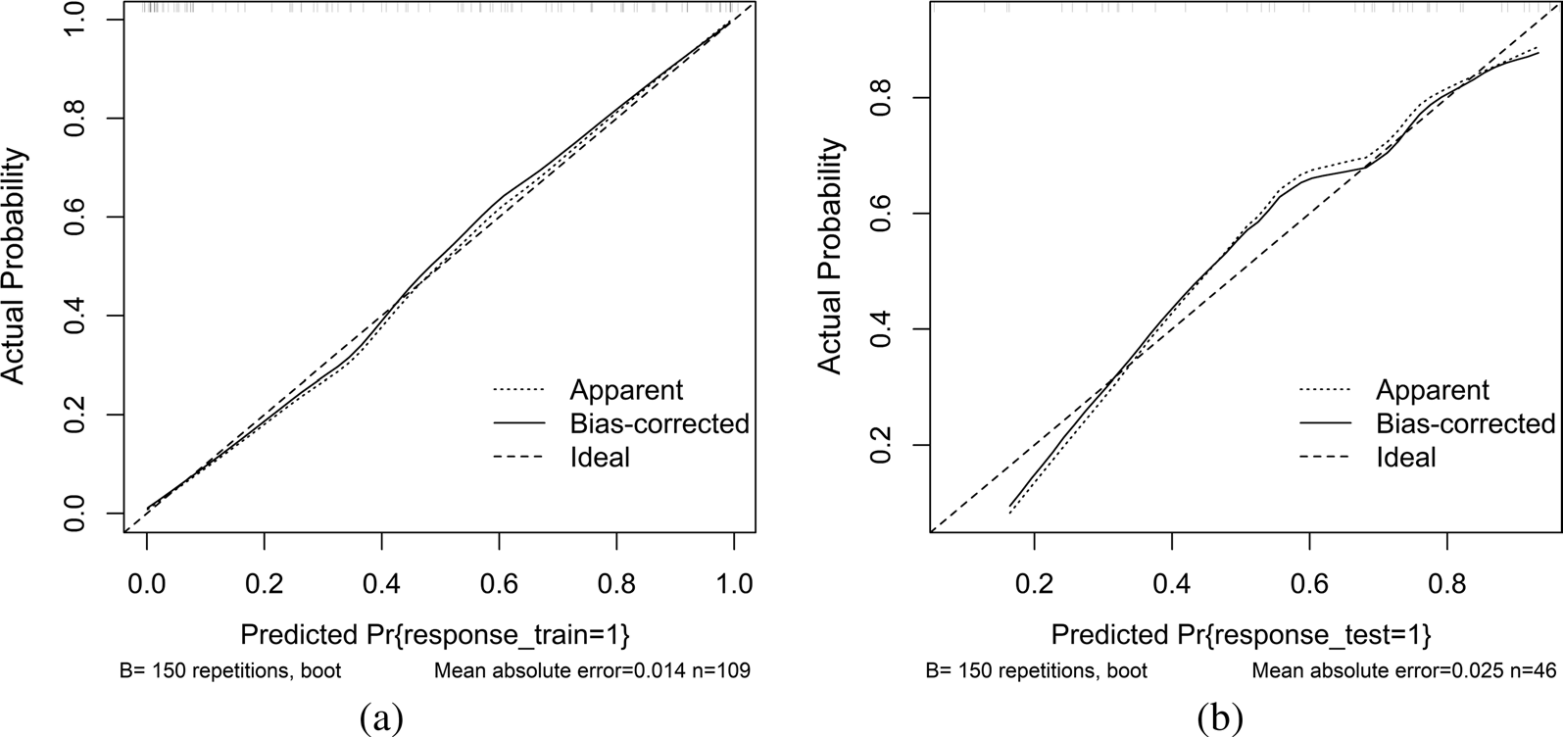
Calibration curves of the nomogram for the training cohort (**a**) and test cohort (**b**)

**Fig. 7:**
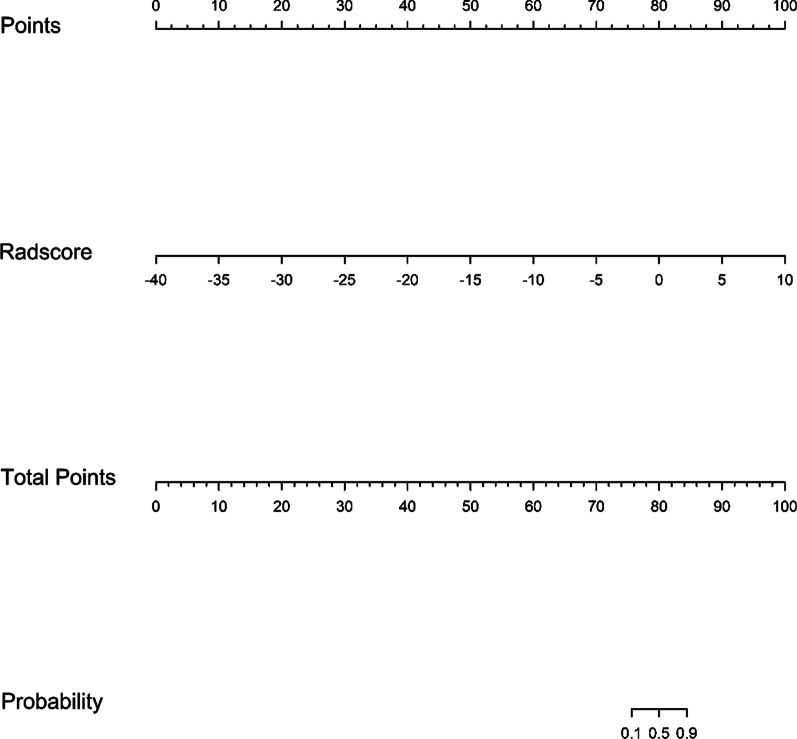
The nomogram of the constructed model. We drew a vertical line from the “Radscore” predictor to the "Total Points" to obtain the score of the predictor. Then, a vertical line was drawn from the "Total Points" to the “probability” axis. Finally, the “probability” value obtained was the probability for the risk of myocardial ischaemia

The ACC, AUC, specificity, sensitivity, positive predictive value (PPV), and negative predictive value (NPV) of the diagnostic myocardial ischaemia model with the training cohort were 0.835, 0.914, 0.733, 0.959, 0.957, and 0.746, respectively; with the test cohort, these values were 0.717, 0.827, 0.684, 0.741, 0.650 and 0.769, respectively (Table 2). The ROC curves of the training cohort and test cohort are shown in Fig. 5. The corresponding calibration curves of the model are displayed in Fig. 6 for the training cohort and the test cohort. Figure 7 shows the nomogram of the constructed multivariable logistic regression model, which estimates the risk of myocardial ischaemia according to the calculated Radscore. Every patient had a Radscore value based on the selected 14 features. According to the Radscore and nomogram, we can obtain the probability of each patient's risk of myocardial ischaemia.

**Table 2:** Machine learning model for training cohort and test cohort

	ACC	AUC	Specificity	Sensitivity	PPV	NPV
Train cohort	0.835	0.914	0.733	0.959	0.957	0.746
Test cohort	0.717	0.827	0.684	0.741	0.650	0.769

### Statistical power calculation

The patients were randomly divided at a ratio of 7:3. There were 60 positive and 49 negative patients in the training cohort and 19 positive and 27 negative patients in the test cohort. The ratio of the training cohort to the test cohort is close to 1:1, at which the logical regression model performs well. The event-per-predictor ratio was 7.78 since there were a total of 14 predictors in our model. An event-per-predictor ratio greater than 5 is recommended in logistic regression models according to the rule of thumb [16]. Therefore, we believe that there are no major concerns regarding model overfitting.

In our study, the sample sizes in the training cohort were ${n}_{A}=60$ and ${n}_{B}=49$, with feature means of ${\mu }_{A}=-3.25$ and ${\mu }_{B}=1.71$, respectively, and a standard deviation of $\sigma =5.024$. The sample sizes in the test cohort were ${N}_{A}=19$ and $N=27$. The two-sided significance level was $\alpha$ = 0.05, and we obtained a mean statistical power of $1-\beta$ = 0.895.

## Discussion

This proof-of-concept study shows that the combination of radiomics and machine learning algorithms can help in the diagnosis of myocardial ischaemia from CCTA images. The extracted information quantifying the spatial and textural properties of CCTA images may generally be invisible to the naked eye. The constructed imaging biomarkers have the potential to objectify our interpretation of CCTA images and increase the diagnostic accuracy for myocardial ischaemia.

At present, the gold standard for myocardial ischaemia diagnoses consists of stress tests, such as stress SPECT/PET or stress CMR. However, stress tests may pose potential risks to patients with suspected CHD. Clinically, myocardial ischaemia is conventionally diagnosed based on comprehensive information including the patient’s history, clinical characteristics, and electrocardiography. In this article, we grouped patients based on SPECT findings and clinical diagnoses.

CCTA is a non-invasive vascular imaging technique commonly used to observe coronary narrowness and plaques from anatomical structures and is routinely used to track suspicious CHD. Many methods also exist for evaluating coronary function based on CCTA, such as CT blood FFR-CT [17–20], coronary calcium score [21] and perivascular fat [22]. Many qualitative imaging markers identified by CCTA [23, 24] were found to predict subsequent major adverse cardiac events (MACEs) [25, 26]. However, because of the properties of these markers, they tend to change based on the observation room and the observer. There is little direct information about ischaemia of the myocardium [27].

Radiomics offers a large number of high-throughput mathematical objectives for describing different lesion characteristics, especially parameters that are invisible to the naked eye or cannot be quantified through routine analysis, such as texture and shape. It has been widely reported that radiomics can provide more information on the diagnosis and prognosis of various diseases than conventional approaches [11–13]. In this study, patients in the myocardial ischaemia group were diagnosed by stress SPECT scans, and the patients in the normal myocardial blood supply group had no clinical signs of myocardial ischaemia. The CCTA myocardial radiomics features of all patients were extracted, and feature selection and model construction were performed under the guidance of the diagnosis results from stress SPECT and/or clinical information. Therefore, we believe that the constructed model has the potential to predict myocardial ischaemia. Additionally, the evaluation index was used to verify the effectiveness of the model. Myocardial ischaemia may lead to changes in myocardial function and tissues, which may not be recognized by the naked eye at an early stage. However, these subtle changes could be discovered by using high-throughput radiomics features. This may be the reason why CCTA myocardial radiomics features can be used to predict myocardial ischaemia. Wenchao Hu et al. [28] extracted 1409 radiomics features from target lesions (lesions with a tendency to cause myocardial ischaemia predicted by FFR measurements) on CCTA images. The AUCs of the training and test cohort in predicting myocardial ischaemia were 0.762 and 0.671, respectively. According to the results of our experiment, the AUCs of the training and test cohort are 0.914 and 0.827, respectively. The higher AUCs may be due to the inclusion of more patients in our study. In addition, the radiomics features of the myocardium may have a greater ability to reflect myocardial ischaemia than those of target lesions. Our results are consistent with other findings reported in the literature; ZhenYu Shu et al. utilized myocardial radiomics features to recognize chronic myocardial ischaemia [29], with AUCs of 0.839 and 0.816 for the training and test cohort, respectively. This work adds further evidence that radiomics has the potential to identify myocardial ischaemia.

One of the characteristics of this paper is the use of deep learning technology for the segmentation of the myocardium, which decreases the subjective differences among doctors and reduces the duration of manual analysis. In addition, three-dimensional segmentation allows an overall assessment of the myocardium. Furthermore, a large number of extracted high-order features provide multidimensional parameters for the diagnosis of ischaemia by CCTA.

This article has some limitations that should be acknowledged. First, the size of the data is small, despite the average statistical power of 0.895, and the generalizability of the results still needs to be validated with an external, central dataset. Second, we excluded patients with other heart diseases to reduce their impact on the model. However, this may have led to potential selection bias, which should be considered in subsequent studies. Third, the model incorporates only the radiomics features from CCTA images; clinical indicators could be further combined to increase the effectiveness of the diagnosis. Since the main focus of this paper was to explore the effectiveness of CCTA, the value of combining clinical indicators will be explored in a future study. Finally, this study is limited to the prediction of myocardial ischaemia, and in future studies, we will further explore the value of myocardial textures in predicting other conditions such as heart failure and heart deposits.

## Conclusion

In conclusion, this paper develops a multivariable logic regression model based on CCTA images for diagnosing myocardial ischaemia. These results will add value to CCTA research and facilitate a more accurate diagnosis for these patients.

## Supplementary Information

**Additional file 1**: Table 1. The contrast media injection protocol.

**Additional file 2**: AK software radiomics parameter description.

## Data Availability

The datasets used and/or analysed during the current study are available from the corresponding author on reasonable request.

## Abbreviations

CCTA: Coronary computed tomography angiography
LASSO: Least absolute shrinkage and selection operator
CHD: Coronary atherosclerotic heart disease
SPM: Spearman correlation analysis
ROC: Receiver operating characteristic
AUC: Area under the curve
PPV: Positive predictive value
NPV: Negative predictive value
