# Supplementary material for: Exploring the diagnostic effectiveness for myocardial ischaemia based on CCTA myocardial texture features
Source: BMC Cardiovasc Disord. 2021 Aug 31;21:416. doi: 10.1186/s12872-021-02206-z (PMC8406838; doi:10.1186/s12872-021-02206-z)
Supplement: Supplementary file 1 — Additional file 1: Table 1. The contrast media injection protocol. [file 12872_2021_2206_MOESM1_ESM.pdf]

Table 1 The contrast media injection protocol*

BMI	Weight					BMI	Weight				
	<50 kg	50~60 kg	60~70 kg	70~80 kg	>80 kg		65~70 kg	70~75 kg	75~80 kg	80~90 kg	>90 kg
BMI<19			3.0 ml/s			27≦BMI	4.6 ml/s	4.8 ml/s	5.2 ml/s	5.5 ml/s	5.8 ml/s
19≦BMI<23	3.5 ml/s	3.5 ml/s	3.8 ml/s	4.0 ml/s	4.0 ml/s						
23≦BMI<27	3.5 ml/s	3.8 ml/s	4.3 ml/s	5.0 ml/s	5.2 ml/s						

*The duration of coronary injection was 12s. When the heart rate was increased by 5 beats/min on the basis of 75 beats/min, the injection rate was added 0.2ml/s, and the dosage was also increased. The rate of saline was the same as the injection rate of contrast agent, and the dosage was 30 ml.
