# Supplementary material for: Exploring the diagnostic effectiveness for myocardial ischaemia based on CCTA myocardial texture features
Source: BMC Cardiovasc Disord. 2021 Aug 31;21:416. doi: 10.1186/s12872-021-02206-z (PMC8406838; doi:10.1186/s12872-021-02206-z)
Supplement: Supplementary file 2 — Additional file 2: AK software radiomics parameter description. [file 12872_2021_2206_MOESM2_ESM.pdf]

# AK Software

# Radiomics Parameters Description

GE Life Sciences

AA R&D Team

February 19th, 2017
Guangzhou, China

## Contents Guide


**1. Histogram Parameters ........ 4**
- **1.1 Energy: ........ 4**
- **1.2 Entropy: ........ 4**
- **1.3 MaxIntensity: ........ 4**
- **1.4 MinIntensity: ........ 4**
- **1.5 MeanValue: ........ 4**
- **1.6 Mean absolute deviation: ........ 4**
- **1.7 MedianIntensity: ........ 5**
- **1.8 Range: ........ 5**
- **1.9 Root mean square (RMS): ........ 5**
- **1.10 Standard deviation: stdDeviation ........ 5**
- **1.11 Uniformity: ........ 5**
- **1.12 Variance: ........ 5**
- **1.13 Volume Count ........ 5**
- **1.14 Voxel Value Sum ........ 5**
- **1.15 RelativeDeviation ........ 5**
- **1.16 Frequency Size ........ 6**
- **1.17 Quantiles ........ 6**
- **1.18 Percentiles ........ 6**
- **1.19 Skewness ........ 7**
- **1.20 Kurtosis ........ 8**

**2. Texture Parameters ........ 9**
- **2.1 Energy ........ 9**
- **2.2 Entropy ........ 9**
- **2.3 Correlation ........ 9**
- **2.4 Inertia ........ 10**
- **2.5 Cluster Shade ........ 10**
- **2.6 Cluster Prominence ........ 10**

**3. Form Factor Parameters ........ 12**
- **3.1 Sphericity: ........ 12**
- **3.2 Surface area: ........ 12**
- **3.3 Compactness 1: ........ 12**
- **3.4 Compactness 2: ........ 12**
- **3.5 Maximum 3D diameter: ........ 12**
- **3.6 Spherical disproportion: ........ 12**
- **3.7 Surfacetovolumeratio: ........ 13**
- **3.8 Volume: ........ 13**
- **3.9 VolumeCC and VolumeMM. ........ 13**

**4. GLCM Parameters ........ 14**
- **4.1 Energy of GLCM ........ 14**
- **4.2 Entropy of GLCM ........ 15**
- **4.3 Inertia of GLCM ........ 15**
- **4.4 Correlation ........ 16**
- **4.5 Inverse Difference Moment ........ 16**
- **4.6 Haralick features ........ 17**
  - 4.6.1 Haralick Correlation ........ 17
  - 4.6.2 Angular Second Moment ........ 18
  - 4.6.3 Contrast ........ 18
  - 4.6.4 Haralick Entropy ........ 18
  - 4.6.5 HaraVariance ........ 18
  - 4.6.6 sumAverage ........ 18

4.6.7 sumVariance ........ 18
4.6.8 sumEntropy ........ 18
4.6.9 differenceVariance ........ 18
4.6.10 differenceEntropy ........ 19
4.6.11 inverseDifferenceMoment ........ 19

**5. RLM Parameters ........ 20**
**5.1 Short Run Emphasis (18 Parameters) ........ 20**
**5.2 Long Run Emphasis (18Parameters) ........ 20**
**5.3 Grey Level Non-uniformity(18Parameters) ........ 20**
**5.4 Run Length Non-uniformity(18Parameters) ........ 20**
**5.5 Low Grey Level Run Emphasis(18Parameters) ........ 21**
**5.6 High Grey Level Run Emphasis(18Parameters) ........ 21**
**5.7 Short Run Low Grey Level Emphasis(18Parameters) ........ 21**
**5.8 Short Run High Grey Level Emphasis(18Parameters) ........ 21**
**5.9 Long Run Low Grey Level Emphasis(18Parameters) ........ 21**
**5.10 Long Run High Grey Level Emphasis(18Parameters) ........ 21**

**6. Reference: ........ 22**

# 1. Histogram Parameters

Histogram parameters are concerned with properties of individual pixels. They describe the distribution of voxel intensities within the CT image through commonly used and basic metrics. Let $X$ denote the three dimensional image matrix with $N$ voxels and $P$ the first order histogram divided by $N_l$ discrete intensity levels. The following first order statistics were extracted:

## 1.1 Energy:

The energy feature measures the uniformity of the intensity level distribution. If the value is high, then the distribution is to a small number of intensity levels. Energy can be defined as:

$$\mathrm{energy} = \sum_{\mathrm{i}}^{\mathrm{N}} \mathrm{X(i)}^2$$

## 1.2 Entropy:

The entropy measures the randomness of the distribution of the coefficients values over the intensity levels. If the value of entropy is high, then the distribution is among more intensity levels in the image. This measurement is the inverse of energy. A simple image has low entropy while a complex image has high entropy. Entropy can be defined as:

$$entropy = -\sum_{i=1}^{N_l} P(i)\, log_2\, P(i)$$

## 1.3 MaxIntensity:

The maximum intensity value of $X$.

## 1.4 MinIntensity:

The minimum intensity value of $X$.

## 1.5 MeanValue:

The mean measures the average value of the intensity values.

$$mean = \frac{1}{N}\sum_{i}^{N} X(i)$$

## 1.6 Mean absolute deviation:

The mean of the absolute deviations of all voxel intensities around the mean intensity value.

## 1.7 MedianIntensity:

The median intensity value of $X$.

## 1.8 Range:

The range of intensity values of $X$.

## 1.9 Root mean square (RMS):

$$RMS = \sqrt{\frac{\sum_i^N X(i)^2}{N}}$$

## 1.10 Standard deviation: stdDeviation

Is a measure that is used to quantify the amount of variation or dispersion of a set of data values.

$$standard\ deviation = \left(\frac{1}{N-1}\sum_{i=1}^{N}(X(i)-\bar{X})^2\right)^{1/2}$$

where $\bar{X}$ is the mean of $X$.

## 1.11 Uniformity:

$$uniformity = \sum_{i=1}^{N_l} P(i)^2$$

## 1.12 Variance:

Is the average of the squared differences from the Mean.

$$variance = \frac{1}{N-1}\sum_{i=1}^{N}(X(i)-\bar{X})^2$$

where $\bar{X}$ is the mean of $X$.

## 1.13 Volume Count

Describe the size of the ROI.

## 1.14 Voxel Value Sum

Represents the Sum calculations for voxels in the ROI.

## 1.15 RelativeDeviation

Let $\bar{X}$ denote the mean of a set of quantities $X_i$ , then the relative deviation is defined by:

$$\frac{\Delta X_i}{\bar{X}} = \frac{|X_i - \bar{X}|}{\bar{X}}$$

## 1.16 Frequency Size

## 1.17 Quantiles

Quantile normalization is a global adjustment method that assumes the statistical distribution of each sample is the same. The normalization is achieved by forcing the observed distributions to be the same and the average distribution, obtained by taking the average of each quantile across samples. They are cut points dividing the range of a probability distribution into contiguous intervals with equal probabilities, or dividing the observations in a sample in the same way.
For a finite population of *N* equally probable values indexed 1, …, *N* from lowest to highest, the *k*-th *q*-quantile of this population can equivalently be computed via the value of:

$$I_p = N\ k/q$$

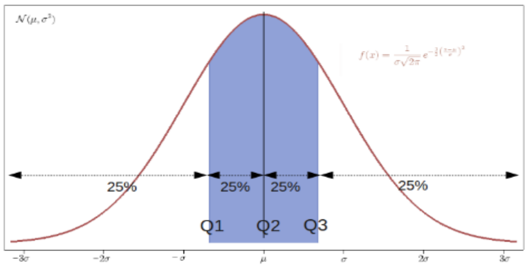


The area below the red curve is the same in the intervals (-∞, *Q1*), (*Q1*, *Q2*), (*Q2*, *Q3*), and (*Q3*, +∞).

In AK software, we have 5 Quantiles:
**Quantile0.025, Quantile0.25, Quantile0.5, Quantile0.75, Quantile0.975.**

## 1.18 Percentiles

A **percentile** (or a **centile**) is a measure used in statistics indicating the value below which a given percentage of observations in a group of observations fall.
The percentile, p%, of a distribution is defined as that value of the brightness *a* such that:
P(a) = p%.
or equivalently: $\int_{-\infty}^{a} P(\alpha) = p\%$
The P-th percentile $0 < P \leq 100$ of a list of N ordered values (sorted from least to greatest) is the smallest value in the list such that P percent of the data is less than or equal to that value. This is obtained by first calculating the ordinal rank and then taking the value from the ordered list that corresponds to that rank. The ordinal rank *n* is calculated using this formula

$$n = \frac{P}{100} * N$$

AK Software have 19 Percentiles.
**Percentile5, Percentile10, Percentile15, Percentile20, Percentile25, Percentile30, Percentile35, Percentile40, Percentile45, Percentile50, Percentile55, Percentile60, Percentile65, Percentile70, Percentile75, Percentile80, Percentile85, Percentile90, Percentile95**

## 1.19 Skewness

Represents the degree of asymmetric distribution in the image histogram, this means that in some distribution of data, the right and the left of the distribution are perfect mirror images of one another, the mean, median and mode are all measures of the center of a set of data. The Skewness of the data can be determined by how these quantities are related to one another. [6]

High values of Skewness means that the distribution is asymmetric otherwise the image is more symmetric; negative skew is when the numerical distribution is relatively long also called negative Skewness distribution, the opposite is referred as positive Skewness distribution (positive skew). Its possible to use The positive and negative Skewness to draw comparisons between the uniform distribution curve.

Formula:

$$skewness = \frac{\frac{1}{N}\sum_{i=1}^{N}(X(i)-\bar{X})^3}{\left(\sqrt{\frac{1}{N}\sum_{i=1}^{N}(X(i)-\bar{X})^2}\right)^3}$$

where $\bar{X}$ is the mean of $X$.

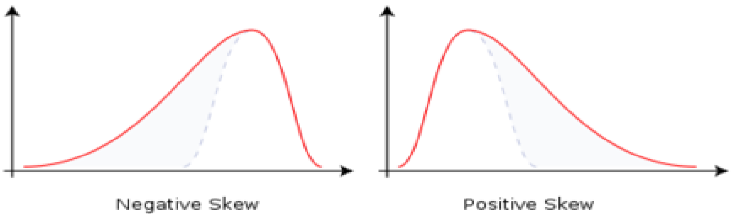

## 1.20 Kurtosis

Kurtosis is a measure of whether the data are heavy-tailed or light-tailed relative to a normal distribution. That is, data sets with high kurtosis tend to have heavy tails, or outliers. Data sets with low kurtosis tend to have light tails, or lack of outliers. A uniform distribution would be the extreme case. [3]
When Kurtosis have small values, it shows more concentration, in contrast when the Kurtosis is bigger is more dispersed. Usually the size of positive and negative kurtosis is compared with the normal distribution curve. Positive Kurtosis indicates that the normal distribution curve is more smooth, on the other hand, the negative Kurtosis indicates that the normal distribution is more precipitous.

Kurtosis is unfortunately harder to picture than Skewness, but the illustrations below, should help. All of these three distributions have **mean of 0, standard deviation of 1, and Skewness of 0**, and all are plotted on the same horizontal and vertical scale**. Look at the progression from left to right, as kurtosis increases.** [3]

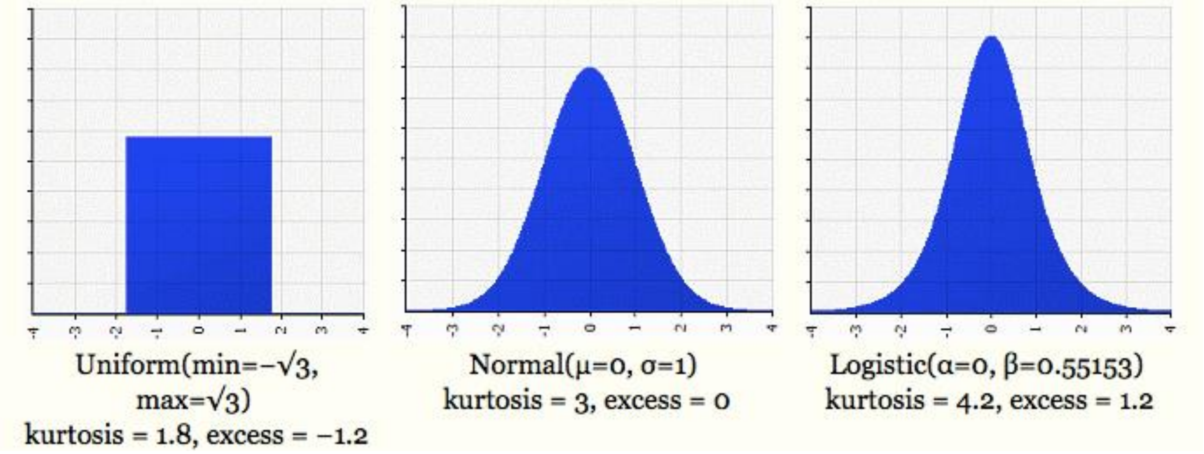


Image illustration of Kurtosis [4]

Formula:

$$kurtosis = \frac{\frac{1}{N}\sum_{i=1}^{N}(X(i)-\bar{X})^4}{\left(\sqrt{\frac{1}{N}\sum_{i=1}^{N}(X(i)-\bar{X})^2}\right)^2}$$

where $\bar{X}$ is the mean of $X$.

## 2. Texture Parameters

Texture is one of the important characteristics used in identifying objects or regions of interest in an image, texture represents the appearance of the surface and how its elements are distributed. It is considered an important concept in machine vision, in a sense it assists in predicting the feeling of the surface (e.g. smoothness, coarseness …etc.) from image. Various texture analysis approaches tend to represent views of the examined textures form different perspectives, and due to multi-dimensionality of perceived texture, there is not an individual method that can be sufficient for all textures. Therefore, AK software is mainly concerned with texture classification accuracy improvement using textures features statistical based methods.

### 2.1 Energy

This feature Returns the sum of squared elements in the GLCM. Range = [0 1]
Energy is 1 for a constant image. Is high when image has very good homogeneity or when pixels are very similar The Property Energy is also known as uniformity, uniformity of energy, and angular second moment. [2]

**Formula:**

$$\sum_{i,j} g(i,j)^2$$

*g is a GLCM
Where i,j are the spatial coordinates of g (i,j).

### 2.2 Entropy

This is a measure of randomness, having its highest value when the elements of **g** are all equal. In the case of a checkerboard, the entropy would be low.

Formula

$$-\sum_{i,j} g(i,j)\log_2(i,j)$$

### 2.3 Correlation

Correlation measures the linear dependency of grey levels of neighboring pixels, in other words, it measures the similarity of the grey levels in neighboring pixels, tells how correlated a pixel is to its neighbor over the whole image. [2]
Range = [-1 1]. Correlation is 1 or -1 for a perfectly positively or negatively correlated image.

Formula

$$-\sum_{i,j}\frac{(i-\mu)(j-\mu)g(i,j)}{\sigma^2}$$

## 2.4 Inertia

It reflects the clarity of the image and texture groove depth. The contrast is proportional to the texture groove, high values of the groove produces more clarity, in contrast small values of the groove will result in small contrast and fuzzy image. [2]

**Formula**

$$\sum_{i,j} ((i-j)^2 g(i,j))$$

## 2.5 Cluster Shade

**Cluster analysis** or **clustering** is the task of grouping a set of objects in such a way that objects in the same group (**cluster**) are more similar (in some sense or another) to each other than to those in other groups (clusters). It is a common technique for statistical data analysis.

Cluster Shade in clustered shading, we group similar view samples according to their position and, optionally, normal into clusters.

In AK Software we have the 36 parameters related to Cluster analysis, first we describe the 18 related to Cluster Shade.

**(ClusterShade_AllDirection_offset1, ClusterShade_AllDirection_offset1_SD, ClusterShade_angle0_offset1, ClusterShade_angle45_offset1, ClusterShade_angle90_offset1, ClusterShade_angle135_offset1, ClusterShade_AllDirection_offset4, ClusterShade_AllDirection_offset4_SD, ClusterShade_angle0_offset4, ClusterShade_angle45_offset4, ClusterShade_angle90_offset4, ClusterShade_angle135_offset4, ClusterShade_AllDirection_offset7, ClusterShade_AllDirection_offset7_SD, ClusterShade_angle0_offset7, ClusterShade_angle45_offset7, ClusterShade_angle90_offset7, ClusterShade_angle135_offset7).**

Formula

$$\sum_{i,j} ((i-\mu)+(j-\mu))^3 g(i,j)$$

## 2.6 Cluster Prominence

Cluster Prominence is a measure of asymmetry of a given distribution, high values of this feature indicate that the symmetry of the image is low, in medical imaging low values of cluster prominence represent a smaller peak for the image grey level value and usually the grey level difference between the forms is small.

**(ClusterProminence_AllDirection_offset1, ClusterProminence_AllDirection_offset1_SD,**
**ClusterProminence_angle0_offset1,**
**ClusterProminence_angle45_offset1,**
**ClusterProminence_angle90_offset1,**
**ClusterProminence_angle135_offset1,**
**ClusterProminence_AllDirection_offset4,**
**ClusterProminence_AllDirection_offset4_SD,**
**ClusterProminence_angle0_offset4,**
**ClusterProminence_angle45_offset4,**
**ClusterProminence_angle90_offset4,**
**ClusterProminence_angle135_offset4,**
**ClusterProminence_AllDirection_offset7,**
**ClusterProminence_AllDirection_offset7_SD,**
**ClusterProminence_angle0_offset7,**
**ClusterProminence_angle45_offset7,**

**ClusterProminence_angle90_offset7, ClusterProminence_angle135_offset7)**

## Formula

$$\sum_{i,j}((i-\mu)+(j-\mu))^4 g(i,j)$$

## 3. Form Factor Parameters

These group of features includes descriptors of the three-dimensional size and shape of the tumor region. Let in the following definitions $V$ denote the volume and $A$ the surface area of the volume of interest. We determined the following shape and size based features:

### 3.1 Sphericity:

$$sphericity = \frac{\pi^{\frac{1}{3}}(6V)^{\frac{2}{3}}}{A}$$

### 3.2 Surface area:

The surface area is calculated by triangulation (i.e. dividing the surface into connected triangles) and is defined as:

$$A = \sum_{i=1}^{N} \frac{1}{2} |a_i b_i \times a_i c_i|$$

### 3.3 Compactness 1:

$$compactness\ 1 = \frac{V}{\sqrt{\pi} A^{\frac{2}{3}}}$$

### 3.4 Compactness 2:

$$compactness\ 2 = 36\pi \frac{V^2}{A^3}$$

### 3.5 Maximum 3D diameter:

The maximum three-dimensional tumor diameter is measured as the largest pairwise Euclidean distance, between voxels on the surface of the tumor volume.

### 3.6 Spherical disproportion:

$$spherical\ disproportion = \frac{A}{4\pi R^2}$$

Where $R$ is the radius of a sphere with the same volume as the tumor.

Where $N$ is the total number of triangles covering the surface and $a$, $b$ and $c$ are edge vectors of the triangles.

### 3.7 Surfacetovolumeratio:

$$surface\ to\ volume\ ratio = \frac{A}{V}$$

### 3.8 Volume:

The volume ($V$) of the tumor is determined by counting the number of pixels in the tumor region and multiplying this value by the voxel size.

### 3.9 VolumeCC and VolumeMM.

The maximum 3D diameter, surface area and volume provide information on the size of the lesion. Measures of compactness, spherical disproportion, sphericity and the surface to volume ratio describe how spherical, rounded, or elongated the shape of the tumor is.

# 4. GLCM Parameters

The Grey level co-occurrence matrix (GLCM) $\mathbf{P}\,(\mathbf{i},\mathbf{j}|\boldsymbol{\theta},\mathbf{d})$ represents the joint probability of certain sets of pixels having certain grey-level values. It calculates how many times a pixel with grey-level **i** occurs jointly with another pixel having a grey value **j**. By varying the displacement vector **d** between each pair of pixels.

The advantage of the co-occurrence matrix calculations is that the co-occurring pairs of pixels can be spatially related in various orientations with reference to distance and angular spatial relationships, as on considering the relationship between two pixels at a time. As a result, the combination of grey levels and their positions are exhibited apparently. Therefore, it is defined as “A two dimensional histogram of gray levels for pair of pixels, which are separated by a fixed spatial relationship”. However, the matrix is sensitive to rotation. With the change of different offsets define pixel relationships by varying directions.

The rotation angle of an offset: $0°, 45°, 90°, 135°$ and displacement vectors (distance to the neighbor pixel: 1, 2, 3 ...), different co-occurrence distributions from the same image of reference. GLCM of an image is computed using displacement vector d defined by its radius, (distance or count to the next adjacent neighbor preferably is equal to one) and rotational angles.

Example:

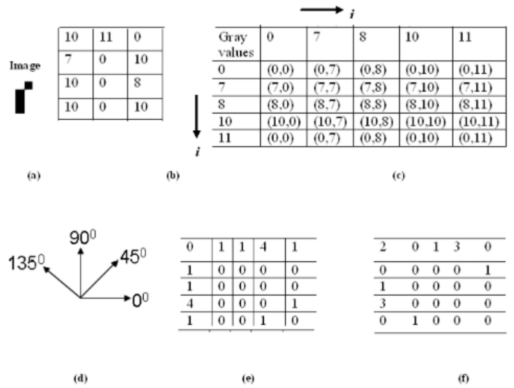


**Fig. 1 (a) test image; (b) test image intensity values in matrix form; (c) generalized fromof GLCM of test image; (d) roation offsets defines the pixel spatial relationships;(e) and (f) GLCMs of the image at an angle of $0^0$ and $45^0$.**

## 4.1 Energy of GLCM

This feature Returns the sum of squared elements in the GLCM. Range = [0 1]
Energy is 1 for a constant image. Is high when image has very good homogeneity or when pixels are very similar The Property Energy is also known as uniformity, uniformity of energy, and angular second moment.

In AK Software we have 18 parameters related to the GLCM Energy : **GLCMEnergy_AllDirection_offset1**, **GLCMEnergy_AllDirection_offset1_SD**, **GLCMEnergy_angle0_offset1**, **GLCMEnergy_angle45_offset1**, **GLCMEnergy_angle90_offset1**, **GLCMEnergy_angle135_offset1**, **GLCMEnergy_AllDirection_offset4**, **GLCMEnergy_angle0_offset4**, **GLCMEnergy_angle45_offset4**, **GLCMEnergy_angle90_offset4**,

**GLCMEnergy_angle135_offset4, GLCMEnergy_AllDirection_offset4_SD, GLCMEnergy_AllDirection_offset7, GLCMEnergy_angle0_offset7, GLCMEnergy_angle45_offset7, GLCMEnergy_angle90_offset7, GLCMEnergy_angle135_offset7, GLCMEnergy_AllDirection_offset7_SD**

**(Formula)**

$$\sum_{i,j} g(i,j)^2$$

*g is a GLCM

Where i,j are the spatial coordinates of g (i,j).

## 4.2 Entropy of GLCM

Entropy is a measure of randomness of intensity image.

Entropy shows the amount of information of the image that is needed for the image compression. Entropy measures the loss of information or message in a transmitted signal and also measures the image information.

In AK Software we have the 18 parameters related to the GLCM Entropy (**GLCMEntropy_AllDirection_offset1, GLCMEntropy_AllDirection_offset1_SD, GLCMEntropy_angle0_offset1, GLCMEntropy_angle45_offset1, GLCMEntropy_angle90_offset1, GLCMEntropy_angle135_offset1, GLCMEntropy_AllDirection_offset4,GLCMEntropy_AllDirection_offset4_SD, GLCMEntropy_angle0_offset4, GLCMEntropy_angle45_offset4, GLCMEntropy_angle90_offset4, GLCMEntropy_angle135_offset4, GLCMEntropy_AllDirection_offset7, GLCMEntropy_AllDirection_offset7_SD, GLCMEntropy_angle0_offset7, GLCMEntropy_angle45_offset7, GLCMEntropy_angle90_offset7, GLCMEntropy_angle135_offset7**)

Formula

$$-\sum_{i,j} g(i,j)\log_2(i,j)$$

## 4.3 Inertia of GLCM

It reflects the clarity of the image and texture groove depth. The contrast is proportional to the texture groove, high values of the groove produces more clarity, in contrast small values of the groove will result in small contrast and fuzzy image.

In AK Software we have the 18 parameters related to the Inertia (**Inertia_AllDirection_offset1, Inertia _AllDirection_offset1_SD, Inertia _angle0_offset1, Inertia _angle45_offset1, Inertia _angle90_offset1, Inertia _angle135_offset1, Inertia _AllDirection_offset4, Inertia _AllDirection_offset4_SD, Inertia _angle0_offset4, Inertia _angle45_offset4, Inertia _angle90_offset4, Inertia _angle135_offset4, Inertia _AllDirection_offset7, Inertia _AllDirection_offset7_SD, Inertia _angle0_offset7, Inertia _angle45_offset7, Inertia _angle90_offset7, Inertia _angle135_offset7**)

**Formula**

$$\sum_{i,j}((i-j)^2 g(i,j))$$

## 4.4 Correlation

Image-based
Correlation measures the similarity of the grey levels in neighboring pixels, tells how correlated a pixel is to its neighbor over the whole image.
Range = [-1 1]. Correlation is 1 or -1 for a perfectly positively or negatively correlated image.

In AK Software we have the 18 parameters related to the Correlation
(**Correlation_AllDirection_offset1**, **Correlation _AllDirection_offset1_SD**,
**Correlation _angle0_offset1**, **Correlation _angle45_offset1**,
**Correlation _angle90_offset1**, **Correlation _angle135_offset1**,
**Correlation _AllDirection_offset4**, **Correlation _AllDirection_offset4_SD**,
**Correlation _angle0_offset4**, **Correlation _angle45_offset4**,
**Correlation _angle90_offset4**, **Correlation _angle135_offset4**,
**Correlation _AllDirection_offset7**, **Correlation _AllDirection_offset7_SD**,
**Correlation _angle0_offset7**, **Correlation _angle45_offset7**,
**Correlation _angle90_offset7**, **Correlation _angle135_offset7**)

Formula

$$-\sum_{i,j}\frac{(i-\mu)(j-\mu)g(i,j)}{\sigma^2}$$

## 4.5 Inverse Difference Moment

Inverse Difference Moment (IDM) is the local homogeneity. It is high when local gray level is uniform and inverse GLCM is high. IDM weight value is the inverse of the Contrast weight.

Formula

$$\sum\sum\frac{1}{1+(\mathbf{i}-\mathbf{j})^2}\mathbf{g}(\mathbf{i},\mathbf{j})$$

## 4.6 Haralick features

$P_{ij} = P(i,j)$ = matrix of relative frequencies with which two neighboring resolution cells separated by distance $d$ occur on the image,
$p(i,j) = P(i,j)/R$ = (i,j)th entry in a normalized gray-tone spatial dependence matrix,
$N_g$ = number of distinct gray levels in the quantized image (the *EBImage haralick.nbins* parameter in the function *computerFeatures.haralick* defaults to 32 gray levels),
$R$ = normalizing constant = number of neighboring resolution cell pairs used in computing a particular gray-tone spatial-dependence matrix.

$R = \sum_{i=1}^{N_g} \sum_{j=1}^{N_g} P(i,j)$ = sum of all elements of co-occurrence frequency matrix

$p(i,j) = \frac{P(i,j)}{R}$ = co-occurence probability matrix

$p_x(i) = \sum_{j=1}^{N_g} p(i,j)$ = i-th entry in the marginal-probability matrix obtained by summing the rows of $p(i,j)$.

$p_y(j) = \sum_{i=1}^{N_g} p(i,j)$ = j-th entry in the marginal-probability matrix obtained by summing the columns of $p(i,j)$.

$$p_{x+y}(k) = \sum_{i=1}^{N_g} \sum_{j=1}^{N_g} \delta_{i+j,k} p(i,j), \qquad k = 2, 3, \ldots, 2N_g$$

$$p_{x-y}(k) = \sum_{i=1}^{N_g} \sum_{j=1}^{N_g} \delta_{|i-j|,k} p(i,j), \qquad k = 0, 1, \ldots, N_g - 1$$

### 4.6.1 Haralick Correlation

Measures the degree of similarity of the gray level of the image in the row or column direction. Represents the local grey level correlation, the greater its value, the greater the correlation;

**(HaralickCorrelation_AllDirection_offset1, HaralickCorrelation_AllDirection_offset1_SD,
HaralickCorrelation_angle0_offset1,
HaralickCorrelation_angle45_offset1,
HaralickCorrelation_angle90_offset1,
HaralickCorrelation_angle135_offset1,
HaralickCorrelation_AllDirection_offset4,
HaralickCorrelation_AllDirection_offset4_SD,
HaralickCorrelation_angle0_offset4,
HaralickCorrelation_angle45_offset4,
HaralickCorrelation_angle90_offset4,
HaralickCorrelation_angle135_offset4,
HaralickCorrelation_AllDirection_offset7,
HaralickCorrelation_AllDirection_offset7_SD,
HaralickCorrelation_angle0_offset7,
HaralickCorrelation_angle45_offset7,
HaralickCorrelation_angle90_offset7,
HaralickCorrelation_angle135_offset7)**

Formula

$$-\sum_{i,j} \frac{(i,j)g(i,j) - \mu_t^2}{\sigma_t^2}$$

* where $\mu_t$ and $\sigma_t$ are the mean and standard deviation of the row (or column, due to symmetry) sums.

### 4.6.2 Angular Second Moment

$$f_1 = \sum_{i=1}^{N_g}\sum_{j=1}^{N_g}\left(\frac{P(i,j)}{R}\right)^2 = \sum_i\sum_j p(i,j)^2$$

### 4.6.3 Contrast

The contrast feature, is a difference moment of the P matrix and is a measure of the contrast or the amount of local variations present in the image.

$$f_2 = \sum_{k=0}^{N_g-1} k^2 \left\{ \sum_{i=1}^{N_g}\sum_{j=1}^{N_g} \delta_{|i-j|,k} p(i,j) \right\} = \sum_{k=0}^{N_g-1} k^2 p_{x-y}(k)$$

### 4.6.4 Haralick Entropy

$$f_9 = -\sum_{i=1}^{N_g}\sum_{j=1}^{N_g} p(i,j)\log(p(i,j))$$

### 4.6.5 HaraVariance

$$f_4 = \sum_{i=1}^{N_g}\sum_{j=1}^{N_g}(i-\mu)^2 p(i,j)$$

### 4.6.6 sumAverage

$$f_6 = \sum_{i=2}^{2N_g} i p_{x+y}(i)$$

### 4.6.7 sumVariance

$$f_7 = \sum_{i=2}^{2N_g}(i-f_8)^2 p_{x+y}(i)$$

### 4.6.8 sumEntropy

$$f_8 = -\sum_{i=2}^{2N_g} p_{x+y}(i)\log(p_{x+y}(i))$$

### 4.6.9 differenceVariance

$$f_{10} = \text{variance of } p_{x-y}$$

### 4.6.10 differenceEntropy

$$f_{11} = -\sum_{i=0}^{N_g-1} p_{x-y}(i) \log(p_{x-y}(i))$$

### 4.6.11 inverseDifferenceMoment

$$f_5 = \sum_{i=1}^{N_g} \sum_{j=1}^{N_g} \frac{1}{1+(i-j)^2} p(i,j)$$

## 5. RLM Parameters

The grey level run-length matrix (RLM) $\mathbf{P_r(i, j \mid \theta)}$ is defined as the numbers of runs with pixels of gray level *i* and run length *j* for a given direction θ. RLMs is generated for each sample image segment having directions (0°,45°,90° &135°), then the following ten statistical features were derived: short run emphasis, long run emphasis, grey level non-uniformity, run length non-uniformity, Low Grey Level Run Emphasis, High Grey Level Run Emphasis, Short Run Low Grey Level Emphasis, Short Run High Grey Level Emphasis, Long Run Low Grey Level Emphasis and Long Run High Grey Level Emphasis.

### 5.1 Short Run Emphasis (18 Parameters)

**ShortRunEmphasis_AllDirection_offset1,**
**ShortRunEmphasis_AllDirection_offset1_SD,**
**ShortRunEmphasis_angle0_offset1,**
**ShortRunEmphasis_angle45_offset1,**
**ShortRunEmphasis_angle90_offset1,**
**ShortRunEmphasis_angle135_offset1,**
**ShortRunEmphasis_AllDirection_offset4,**
**ShortRunEmphasis_AllDirection_offset4_SD,**
**ShortRunEmphasis_angle0_offset4,**
**ShortRunEmphasis_angle45_offset4,**
**ShortRunEmphasis_angle90_offset4,**
**ShortRunEmphasis_angle135_offset4,**
**ShortRunEmphasis_AllDirection_offset7,**
**ShortRunEmphasis_AllDirection_offset7_SD,**
**ShortRunEmphasis_angle0_offset7,**
**ShortRunEmphasis_angle45_offset7,**
**ShortRunEmphasis_angle90_offset7,**
**ShortRunEmphasis_angle135_offset7**

Formula:

$$SRE(\theta) = \frac{1}{n_r} \sum_{i=1}^{M} \sum_{j=1}^{N} \frac{p(i, j, \theta)}{j^2}$$

### 5.2 Long Run Emphasis (18Parameters)

$$LRE(\theta) = \frac{1}{n_r} \sum_{i=1}^{M} \sum_{j=1}^{N} p(i, j, \theta)\, j^2$$

### 5.3 Grey Level Non-uniformity(18Parameters)

$$GLN(\theta) = \frac{1}{n_r} \sum_{i=1}^{M} (\sum_{j=1}^{N} p(i, j, \theta))^2$$

### 5.4 Run Length Non-uniformity(18Parameters)

$$RLN(\theta) = \frac{1}{n_r} \sum_{j=i}^{N} (\sum_{i=1}^{M} p(i, j, \theta))^2$$

### 5.5 Low Grey Level Run Emphasis(18Parameters)

$$LGRE(\theta) = \frac{1}{n_r} \sum_{j=i}^{N} \sum_{i=1}^{M} \frac{p(i, j, \theta)}{i^2}$$

### 5.6 High Grey Level Run Emphasis(18Parameters)

$$HGRE(\theta) = \frac{1}{n_r} \sum_{j=i}^{N} \sum_{i=1}^{M} p(i, j, \theta) i^2$$

### 5.7 Short Run Low Grey Level Emphasis(18Parameters)

$$SRLGE(\theta) = \frac{1}{n_r} \sum_{j=i}^{N} \sum_{i=1}^{M} \frac{p(i, j, \theta)}{i^2 j^2}$$

### 5.8 Short Run High Grey Level Emphasis(18Parameters)

$$SRHGE(\theta) = \frac{1}{n_r} \sum_{j=i}^{N} \sum_{i=1}^{M} \frac{p(i, j, \theta) i^2}{j^2}$$

### 5.9 Long Run Low Grey Level Emphasis(18Parameters)

$$LRLGE(\theta) = \frac{1}{n_r} \sum_{j=i}^{N} \sum_{i=1}^{M} \frac{p(i, j, \theta) j^2}{i^2}$$

### 5.10 Long Run High Grey Level Emphasis(18Parameters)

$$LRHGE(\theta) = \frac{1}{n_r} \sum_{j=i}^{N} \sum_{i=1}^{M} p(i, j, \theta) i^2 j^2$$

where $n_r$ is the total number of runs and $n_p$ is the number of pixels in the image.

# 6. Reference:

[1] CT-based radiomic signature predicts distant metastasis in lung adenocarcinoma Coroller, Thibaud P. et al. Radiotherapy and Oncology, Volume 114, Issue 3, 345 – 350

[2] P. Mohanaiah, P. Sathyanarayana and L. GuruKumar, *Image Texture Feature Extraction Using GLCM Approach*, International Journal of Scientific and Research Publications, Volume 3, Issue 5, May 2013

[3] *NIST/SEMATECH e-Handbook of Statistical Methods*, http://www.itl.nist.gov/div898/handbook/.

[4] Alnihoud J., "Content-Based Image Retrieval System Based on Self Organizing Map, Fuzzy Color Histogram and Subtractive Fuzzy Clustering," International Arab Journal of Information Technology, vol. 9, no. 5, pp. 452- 458, 2012.

[5] Balanda, Kevin P., and H. L. MacGillivray. 1988.
"Kurtosis: A Critical Review". *The American Statistician* 42(2), 111–119.
My thanks to Karl Ove Hufthammer for drawing this article to my attention.

[6] https://en.wikipedia.org/wiki/Kurtosis

[7] Bland, J.M.; Altman, D.G. (1996). "Statistics notes: measurement error" *BMJ*. **312** (7047): 1654.
